# Datasets for the environmental assessment of an apple value chain including fresh fruits, juice and applesauce from an organic low-input production farm

**DOI:** 10.1016/j.dib.2023.109824

**Published:** 2023-11-19

**Authors:** Samuel Le Féon, Thierry Benezech, Gwenola Yannou-Le Bris, Joël Aubin, Imca Sampers, Damien Herreman, Caroline Pénicaud

**Affiliations:** a Université Paris-Saclay, INRAE, AgroParisTech, UMR SayFood, 91120 Palaiseau, France; b UMR SAS, INRAE, Institut Agro, 65 rue de Saint Brieuc, 35042 Rennes, France; c Univ. Lille, CNRS, INRAE, Centrale Lille, UMET, F-59000 Lille, France; d Research Unit VEG-i-TEC, Department of Food Technology, Safety and Health, Faculty of Bioscience Engineering, Ghent University, Campus Kortrijk, Sint-Martens-Latemlaan 2B, 8500 Kortrijk, Belgium; e Les Vergers de Méteren, 59270 Méteren, France

**Keywords:** Life cycle assessment, Apple value chain, Organic production, Life Cycle Inventory

## Abstract

Due to societal concerns, assess the environmental impacts, address the issues and provide labelling to the consumer are growing issues for the agri-food sector. In this context, provide datasets specific to alternative systems is crucial to be able to take into account the variability between systems then address their issues and label them appropriately. This data paper compiles all the data used to produce the life cycle assessment (LCA) environmental of an organic low-input apple value chain including the cultivation of apples at farm, the transformation of a part into juice and applesauce, the retail and the consumption stages. The raw data have mostly been obtained through interviews of the farmer and complemented by literature. They have been used to build a life cycle inventory (LCI), using Agribalyse 3.0 and Ecoinvent 3.8 as background databases. The dataset also compiles the life cycle impact assessment (LCIA) using the characterization method EF3.0. As discussed in an associated scientific paper, this dataset participates in filling two gaps: integrate the variability between systems in the discussion and link upstream (at farm) and downstream (transformation, retail, consuming) impacts. This is done by (1) covering the entire value chain from cradle to grave when most papers found in literature focusses on one stage (e.g. the cultivation of apples) and (2) applying LCA to a system that present specificities not well covered by LCA literature (e.g. low-input cultivation with no fertilization up to now).

Specifications Table

**Table:** 

Subject	Environmental engineering
Specific subject area	Environmental assessment of agri-food value chains
Data format	Raw, analyzed, Filtered
Type of data	Table, CSV
Data collection	Most of the foreground data were collected in face-to-face interviews with the farmer (agricultural activities, transport modes and distances, retail). The means in-out platform was used to calculate LCI related to agricultural activities and sensitivity scenarios for fertilization. They were complemented by technical and scientific literature (energy consumptions for process and storage). Ecoinvent 3.8 and Agribalyse 3.0 were used for background data. LCIA data were calculated using the software Simapro and the characterization method “EF 3.0 method (adapted)”
Data source location	Foreground data collected at farm • Institution: Vergers de Méteren • City/Town/Region: Méteren • Country: France • Latitude and longitude for collected samples/data: N 50°43′53.77′’ / E 2°41′42.565′’ Agribalyse 3.0 • Institution: ADEME • City/Town/Region: Nantes • Country: France • https://agribalyse.ademe.fr/ Ecoinvent 3.8 • Institution: Ecoinvent • City/Town/Region: Zurich • Country: Switzerland • https://ecoinvent.org/ MEANS-InOut • Institution: INRAE and CIRAD • City/Town/Region: Rennes • Country: France https://www6.inrae.fr/means
Data accessibility	Repository name: Inrae Dataverse - https://entrepot.recherche.data.gouv.fr/dataverse/inrae Data identification number: https://doi.org/10.57745/SA0IXW Direct URL to data: https://doi.org/10.57745/SA0IXW
Related research article	Le Féon, S., Benezech, T., Yannou-Le Bris, G., Aubin, J., Sampers, I., Herreman, D., Pénicaud, C., Life cycle assessment of a small-scale and low-input organic apple value chain including fresh fruit, juice and applesauce, Clean. Environ. Syst. 11, 100,141 (2023). https://doi.org/10.1016/j.cesys.2023.100141

## 1 Value of the Data

• The article presents a unique set of LCI and LCIA data of the whole apple value chain including juice and applesauce. It expands the scope of exiting scientific literature, being useful to observe the impacts of the rest of the apple value chain in addition to the – well documented – agricultural step.
• The article presents LCI and LCIA for a small-scale low-input organic apple production rarely studied in literature. It can be useful to scientific community as it relates to a specific context of production. It also increases the knowledge on agricultural systems as Data on sensitivity analysis are provided to help other scientists to put results in perspective. it participates in consider the variability between systems, that can be helpful for scientists as well as for decision makers.
• Scientist and LCA practitioners can benefit from complementary LCI proxies related to missing inputs of organic production for their future models.
• The article can be used to make recommendations for more ecological apple value chains.

## 2 Background

The dataset has been built in support to a research article named: “Life Cycle Assessment of alternative apple value chain including fresh fruits, juice and applesauce”. It aims to compute a life cycle inventory for a specific value chain that is not represented currently in the literature. Therefore, it has the objective to help scientists to better evaluate the environmental impacts of small-scale food value chain from cradle to grave. Specifically, it brings data for small-scale apple production, transformation and distribution. In addition, it provides life cycle impact assessment to feed discussions about alternative systems.

## 3 Data Description

The dataset associated with this article contains files with information on inventory data used for the LCA and the LCIA results presented and discussed in the associated article:

1. (FAIRCHAIN)CS-BEL_raw_data.tab: raw numerical data about: apple production, calibration and storage, juice, applesauce and distribution. These data have been mostly collected face-to-face with the farmer.
2. (FAIRCHAIN)CS-BEL_LCI.tab: LCI for the baseline scenario for the whole value chain.
3. (FAIRCHAIN)CS-BEL_dataset_complementary_LCI.pdf: LCI proxies for inputs missing in databases for organic production (Neem oil, Potassium bicarbonate, Pheromones diffuser) and for the baseline scenario for biowaste treatment.
4. (FAIRCHAIN)CS-BEL_dataset_LCI_for_sensitivity_analysis.pdf: LCI for the sensitivity analysis presented in the associated paper: alternatives scenarios for fertilization, carbon sequestration and apple waste management.
5. (FAIRCHAIN)CS-BEL_dataset_LCI_tables.pdf: LCI summarized in tables
6. (FAIRCHAIN)CS-BEL_dataset_LCIA_apple_value_chain.tab: LCA results calculated by the “EF 3.0 method (adapted)” for the midpoint impact categories: Climate change, Ozone depletion, Ionising radiation, Photochemical ozone formation, Particulate matter, Human toxicity, non-cancer, Human toxicity, cancer, Acidification, Eutrophication, freshwater, Eutrophication, marine, Eutrophication, terrestrial, Ecotoxicity, freshwater, Land use, Water use, Resource use, fossils, Resource use, minerals and metals, Climate change – Fossil, Climate change – Biogenic, Climate change - Land use and LU change, Human toxicity, non-cancer – organics, Human toxicity, non-cancer – inorganics, Human toxicity, non-cancer – metals, Human toxicity, cancer – organics, Human toxicity, cancer – inorganics, Human toxicity, cancer – metals, Ecotoxicity, freshwater – organics, Ecotoxicity, freshwater – inorganics, Ecotoxicity, freshwater – metals.
7. (FAIRCHAIN)CS-BEL_dataset_LCIA_by_product.tab: LCA results for 1 unit of product (i.e. 1 kg of fresh apples at consumer, 1 L bottle of apple juice at consumer and 250 g jar of apple applesauce at consumer).
8. (FAIRCHAIN)CS-BEL_dataset_LCIA_results_of_sensitivity_analysis.tab: Results of the sensitivity analysis.

## 4 Experimental design, materials and methods

### 4.1 LCA methodology

Our study followed ISO 14,040 [1]. Then we applied the four methodological steps of LCA:

- Goal and scope definition (i.e. objectives, geographical and temporal boundaries, functional unit and system boundaries),
- Life Cycle Inventory (LCI),
- Life Cycle Impact Assessment (LCIA),
- Results and interpretation.

Details about those steps are given in the next sections, except for the “results and interpretation” that are the subject of a proper scientific paper [2]. The scientific paper also contains details about the whole LCA.

#### 4.1.1 Goal and scope

The objective of the study is to characterize the impacts related to a low-input organic apple value chain including fresh fruits and derivatives (juice and applesauce) in order to be able to help the farmer to take decisions to improve its system. It declines into sub-objectives: (1) to produce an inventory for an alternative organic production of apples in French Flandres including transformation of part of the production into juice and applesauce, (2) to assess its environmental impacts and evaluate the relative contributions of each stage in order to provide guidance to the farmer for further improvements and (3) to discuss about LCA of alternative systems and to introduce options to test in order to improve the value chain in the future (discussed in the associated paper). Following these objectives, an attributional approach is chosen.

##### 4.1.1.1 System boundaries

**Fig. 1:**
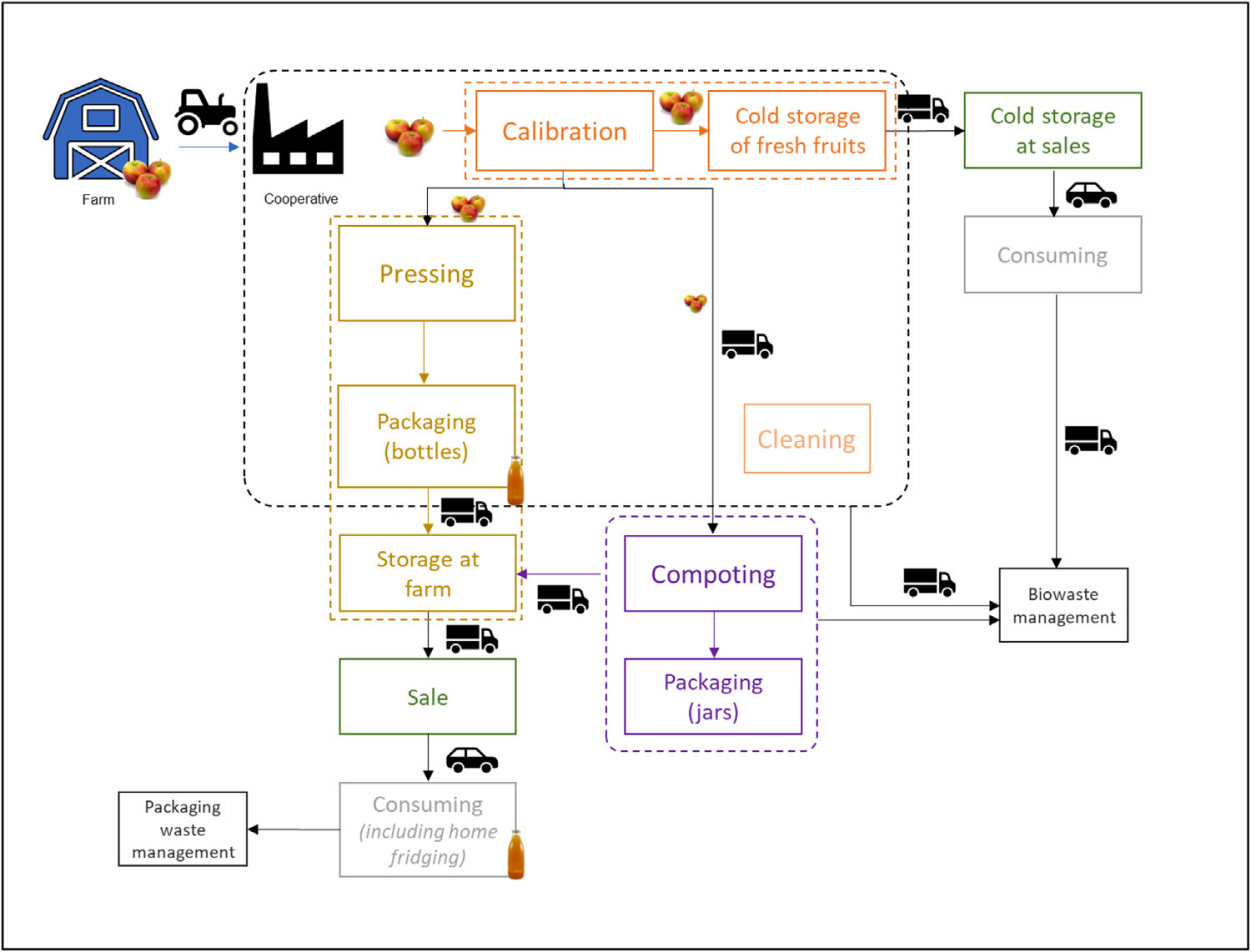
Simplified flow diagram of the studied system from cradle to grave.

This study aims to evaluate the environmental impacts related to the value chain of the apples and its main derivatives (i.e. juice and compote). As the objective is to observe the whole value chain and its specific local organization related, it includes all life cycle stages from cradle to grave (Fig. 1). This whole life cycle is divided into three main sections: agricultural production (from cradle to farm gate), transformation (from farm to retail) and retail and consuming (from retail to grave). The agricultural production includes the cultivation of apples from the tree nursery to the harvest of the apples (including field operations and additional materials). The transformation includes the sorting of fresh fruits (calibration) and their storage until leaving the cooperative to retail. It also includes the transformation of declassed fruits into juice and applesauce (processing and packaging). Finally, the activities from retail to grave includes the different transport stages of the three final products, their retail and their use at consumers (including waste management).

##### 4.1.1.2 Functional unit

The functional unit for this study is defined as “the annual production of fresh apples, juice and applesauce delivered to the consumer”. In addition, focuses on each of the three products have also been made, using mass-based functional units: deliver 1 kg of fresh apples to the consumer, deliver 1 litre of juice to the consumer and deliver 1 kg of applesauce to the consumer. To this purpose and according to standards, subdivision is used when possible (for example, the impacts related to the pressing of apples are 100 % allocated to the juice). When it is not, an input-based allocation is used (for example, the impacts of the cultivation stage are allocated to fresh fruits, juice and applesauce proportionally to the quantity of apples they use as input). These results by mass of product are not the core of this paper so they are not presented in the associated paper but available in this data paper without complete analysis for whatever purpose it may serve.

#### 4.1.2 Life cycle inventory

##### 4.1.2.1 Agricultural stage

Most of the foreground data were collected in face-to-face interview of the farmer. Despite the very high variability involved in such small systems, the objective was to produce a dataset that is representative of one year of production. The surface of the orchard is 3 ha, producing 50 tons of apples a year. The tree density is 1125 trees/ha and the lifespan of the trees is estimated to minimum 25 years. The farmer provided data about mechanical work and inputs. Related emissions to inputs are calculated using Means in-out (https://www6.inrae.fr/means).

Three inputs were absents from databases and proxies were proposed: Neem oil (proxy adapted from [3]), potassium bicarbonate (proxy based on stoichiometry) and pheromones diffusers (proxy using only the major molecule). The proposed inventories are available in (FAIRCHAIN)CS-BEL_dataset_complementary_LCI.pdf. A mix for the management of biowaste in France is also modelled.

Two sensitivity analysis are provided for the cultivation part and discussed in the associated paper:

- Sensitivity scenarios are proposed for fertilization: the same production with a supply of cattle manure in the first year (25 t/ha) and the same production with land preparation and nutrient management derived from [4]. LCI was modelled using Means in-out (https://www6.inrae.fr/means).
- Sensitivity scenarios are proposed for the management of the biomass at orchard (feeding discussion on carbon sequestration.

##### 4.1.2.2 Transformation

After harvesting, apples are transported to a fruit cooperative then calibrated. From 50 tons, 38.45 tons are sold as fresh fruits, the rest is transformed into juice (11.05 tons) and applesauce (0.5 tons). The fresh fruits are stored at the cooperative and progressively sent to retail during the year. In total, 6500 litres of apple juice are produced at the cooperative and 250 kg of applesauce are produced in another workshop. The total water used for process and cleaning was given by the farmer. Amounts of cleaning agents (i.e. sodium hydroxide and nitric acid) were derived from literature [5]. For juice and applesauce productions, 4550 kg and 125 kg of apple pomace are respectively produced and sent to anaerobic digestion. Quantities of products and distances between farm, cooperative and compote workshop were given by the farmer. Details on processes are issued from technical and scientific literature [[6], [7]].

##### 4.1.2.3 Retail and consuming

The farmer gave information about the location where the three products are sold. Fresh fruits are retailed in French organic shops all over the country, but mostly in the production area. Juice and applesauce are locally sold, in a 30 km circle around the farm. Energy consumption for cold storage of fresh fruits at retail is derived from literature [5]. 5 % of fresh fruits are assumed lost at retail, following literature [8]. A proxy inventory representing the French mix for biowaste treatment solutions has been modelled and used ((FAIRCHAIN)CS-BEL_dataset_complementary_LCI.pdf). Distances were calculated in consequence to the farmer assumptions on market and based on literature insights. The share of apples in the total basket were estimated from literature [[8], [9]]. Details are available in (FAIRCHAIN)CS-BEL_raw_data.tab and (FAIRCHAIN)CS-BEL_LCI.tab.

Juice and applesauce are considered to be stored in fridge by the consumer. Electricity consumption is derived from [10]. At consuming, the apple waste is estimated to 25 % of the total mass [11]. A proxy inventory representing the French mix for biowaste treatment solutions has been modelled and used ((FAIRCHAIN)CS-BEL_dataset_complementary_LCI.pdf). Finally, French waste scenarios from Ecoinvent 3.8 were used to model the end-of-life of packaging.

A sensitivity analysis is proposed on the management of apple waste. It is introduced and discussed in the associated paper. Details on the scenarios are provided in (FAIRCHAIN)CS-BEL_dataset_LCI_for_sensitivity_analysis.pdf and results in (FAIRCHAIN)CS-BEL_dataset_LCIA_results_of_sensitivity_analysis.tab.

##### 4.1.2.4 Databases

Agribalyse 3.0 and Ecoinvent 3.8 databases are used for background data.

#### 4.1.3 Life cycle impact assessment

The LCIA was performed using the EF 3.0 (adapted) characterization method [12] and SimaPro 9.3.0.3 [13]. All 16 impact categories of EF were considered: Climate change (CC), Ozone depletion (OD), Ionising radiation (IR), Photochemical ozone formation (POF), Particulate matter (PM), Human toxicity, non-cancer (Tox-nc), Human toxicity, cancer (Tox-c), Acidification (Acid), Eutrophication, freshwater (Eut-F), Eutrophication, marine (Eut-M), Eutrophication, terrestrial (Eut-T), Ecotoxicity, freshwater (Ecotox-F), Land use (LU), Water use (WU), Resource use, fossils (Res-F) and Resource use, minerals and metals (Res-M).

## Limitations

Not applicable.

## Ethics statement

The authors have read and follow the ethical requirements for publication in Data in Brief and confirming that the current work does not involve human subjects, animal experiments, or any data collected from social media platforms.

## CRediT authorship contribution statement

**Samuel Le Féon:** Conceptualization, Formal analysis, Methodology, Investigation, Software, Writing – original draft. **Thierry Benezech:** Investigation, Writing – review & editing. **Gwenola Yannou-Le Bris:** Writing – review & editing. **Joël Aubin:** Conceptualization, Formal analysis, Methodology, Writing – review & editing. **Imca Sampers:** Investigation, Writing – review & editing. **Damien Herreman:** Investigation, Writing – review & editing. **Caroline Pénicaud:** Conceptualization, Formal analysis, Methodology, Writing – review & editing, Supervision.

## Data Availability

LCA_data_Belgium_CS_Small_scale_baseline (Original data) (https://entrepot.recherche.data.gouv.fr/)
